# Lichen nitidus généralisé

**DOI:** 10.11604/pamj.2014.17.32.3595

**Published:** 2014-01-18

**Authors:** Najwa Guerouaz, Badredine Hassam

**Affiliations:** 1 Hôpital Universitaire Ibn Sina, Service de Dermatologie et Vénérologie, Rabat, Maroc

**Keywords:** Lichen nitidus, granulomatose, Lichen nitidus, granulomatosis

## Image en medicine

Le lichen nitidus est une forme inhabituelle de granulomatose idiopathique décrite en 1907 par Pinkus. Il est caractérisé par de multiples petites papules luisantes qui intéressent les organes génitaux, les bras, les avant-bras et le tronc. Un patient âgé de 42 ans, ouvrier spécialisé dans le revêtement des sols présente depuis 2 ans des lésions papuleuses acuminées mesurant 1 à 2 mm de diamètre, de couleur blanche brillante, non prurigineuses intéressant d'abord les avant-bras (A), puis le reste du corps en épargnant le visage (B). La biopsie cutanée a montré un infiltrat lymphohistiocytaire du derme papillaire entre deux crêtes épidermiques effilées donnant l'aspect de “griffe des crêtes” (C). Le lichen nitidus peut survenir à tout âge sans prédominance du sexe. Les papules sont le plus souvent localisées, mais de rares cas ont été rapportés avec une distribution généralisée comme chez notre patient. Les lésions sont habituellement asymptomatiques et guérissent sans séquelles rendant difficile l’évaluation de l'efficacité des thérapeutiques disponibles (UVA, UVB, corticothérapie, astemizole...).

**Figure 1:**
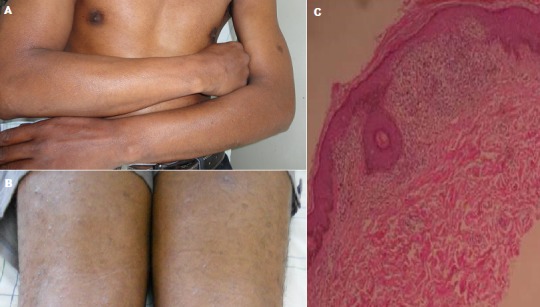
A) Papules luisantes des avant-bras; B) Lésions papuleuse des membres inférieures; C) Infiltrat lymphohistiocytaire du derme papillaire en “griffe des crêtes”

